# Mutation in yl-HOG1 represses the filament-to-yeast transition in the dimorphic yeast *Yarrowia lipolytica*

**DOI:** 10.1186/s12934-023-02161-8

**Published:** 2023-08-16

**Authors:** Dorota A. Rzechonek, Mateusz Szczepańczyk, Aleksandra M. Mirończuk

**Affiliations:** Laboratory for Biosustainability, Institute of Environmental Biology, Wrocław University of Environmental and Life Sciences, Wrocław, Poland

**Keywords:** *Yarrowia lipolytica*, Osmotic stress, Erythritol, Dimorphic transition

## Abstract

**Background:**

*Yarrowia lipolytica* is a dimorphic fungus, which switches from yeast to filament form in response to environmental conditions. For industrial purposes it is important to lock cells in the yeast or filamentous form depending on the fermentation process. yl-Hog1 kinase is a key component of the HOG signaling pathway, responsible for activating the osmotic stress response. Additionally, deletion of yl-Hog1 leads to increased filamentation in *Yarrowia lipolytica*, but causes significant sensitivity to osmotic stress induced by a high concentration of a carbon source.

**Results:**

In this study, we tested the effect of point mutations on the function of yl-Hog1 protein kinase. The targets of modification were the phosphorylation sites (T171A-Y173A) and the active center (K49R). Introduction of the variant HOG1-49 into the *hog1∆* strain partially improved growth under osmotic stress, but did not recover the yeast-like shape of the cells. The HOG1-171/173 variant was not functional, and its introduction further weakened the growth of *hog1∆* strains in hyperosmotic conditions. To verify a genetic modification in filament form, we developed a new system based on green fluorescent protein (GFP) for easier screening of proper mutants.

**Conclusions:**

These results provide new insights into the functions of yl-Hog1 protein in dimorphic transition and constitute a good starting point for further genetic modification of *Y. lipolytica* in filament form.

## Background

*Yarrowia lipolytica* is an unconventional yeast that is a model organism for lipid and polyol metabolism and dimorphism [1–3]. The study of these issues, in addition to advancing our knowledge of fundamental processes, is also important due to the growing biotechnological use of this yeast [4].

Dimorphism means that cells can grow in two forms: as oval cells typical for yeasts, or elongated filaments. *Y. lipolytica* usually grows in the yeast form, but certain environmental conditions or cell damage can cause a dimorphic switch which results in occurrence of filamentous hyphae or pseudohyphal structures [5]. Such a property is both a challenge and an opportunity in biotechnology applications. The filamentous growth is undesirable in many industrial cultures; but on the other hand, the formation of more complex structures might potentially be beneficial in processes that focus on the usage of the entire yeast biomass - such as the production of meat substitutes or animal feed [6].

The second process relevant to both basic science and biotechnology processes is the production of erythritol. Erythritol is a four-carbon polyol that is used as a sweetener in the food industry or in dental care [7, 8]. However, recent studies indicate that its usage might increase the risk of cardiovascular diseases [9]. Thus there is a need for better understanding of metabolism of this polyol in eukaryotic organisms. Erythritol is produced by *Y. lipolytica* and other unconventional yeasts in response to osmotic stress [10–12]. However, it is not a universal response. Formation of erythritol has not been observed in, e.g., the best-studied yeast, *S. cerevisiae*. Despite this difference, many elements of the signaling pathways involved in the stress responses are highly conserved, and the mechanisms described in baker’s yeast have very similar analogues in their unconventional counterparts.

Mobilization of the osmotic stress response in *S. cerevisiae* occurs through a signaling pathway known as HOG (high-osmolarity glycerol), whose central element is the MAPK protein kinase cascade. Kinase Hog1 is the most characteristic and conserved element of the HOG pathway and the last component of the MAPK cascade. Its activation occurs through phosphorylation, catalyzed by the Pbs2 protein kinase. Activated Hog1-P phosphorylates many further downstream elements in the cell, and also enters the cell nucleus, where it affects gene transcription by interacting with transcription factors [13].

The first attempts to characterize yl-Hog1 in *Y. lipolytica* indicated that its knock-out causes a significant increase in sensitivity to osmotic stress, negatively affects erythritol production, and increases filamentation [14]. Since all of these issues may be important for biotechnological applications, we aim to further investigate their connection to the HOG pathway. In this study, we subjected yl-Hog1 protein to point mutations targeting the phosphorylation sites and the enzymatic active center.

*Y. lipolytica* possesses a wide range of molecular tools, but the metabolic engineering of it faces many significant technical difficulties. Due to its thick cell wall, rapid verification of metabolic modifications is troublesome. Moreover, the cell wall in filamentous hyphal form is thicker, which further complicates the procedures. All this made us realize that the existing molecular biological tools and protocols have been developed for *Y. lipolytica* in the yeast form. Moreover, some methods are very useful for genetic modifications aimed at optimizing strains for industrial application, but are not suitable for certain basic studies. An example is the popular technique of incorporating the overexpression cassettes into rDNA sequences that are repeated at multiple locations in the genome [15]. The lack of full control over the site of integration generates some additional variability among the transformants, which can be useful for industry but not for basic research.

In the *Y. lipolytica* genome, there are dozens of sites identified as suitable for integration – these are regions of approximately 5000 bp with no open reading frames or non-coding RNA elements, but surrounded by highly expressed genes [16]. The use of one defined integration site would make it possible to create strains actually differing only by point mutations. However, this would make it necessary to ensure that the cassette does not integrate into an incorrect site through non-homologous recombination – which is a common phenomenon in *Y. lipolytica* [17]. The *hog1∆* strains have a thick cell wall, which made it more difficult to isolate genomic DNA and perform colony PCR. Therefore, screening of correctly inserted transformants was problematic and time-consuming. To address this issue, we introduced a simple reporter system based on green fluorescence protein (GFP).

## Results

### Point mutations in the Hog1 protein

The function of the Hog1 protein depends on the conditions under which its phosphorylation occurs, as well as its ability to phosphorylate subsequent proteins. In the yeast *S. cerevisiae* the site of phosphorylation has been characterized – this is the Thr-X-Tyr motif, where Thr-174 and Tyr-176 are phosphorylated. Mutations in these sites caused a significant increase in sensitivity to osmotic stress [18]. Based on protein similarity, the likely phosphorylation sites in *Y. lipolytica* can be designated as Thr-171 and Tyr-173, as predicted by the UniProt database. In order to test how preventing phosphorylation would affect cell function, a synthetic gene yl-HOG1-171/173 was designed, where the threonine and tyrosine were replaced by alanine (T171A-Y173A mutation). In addition, in *S. cerevisiae*, the enzymatic active center of the Hog1 kinase was also extensively studied. It was found that Lys-52 was crucial for phospho-transfer function. Its substitution for arginine resulted in catalytically inactive kinase [18, 19]. Based on the homology, we repeated this substitution by targeting Lys-49 in yl-HOG1 and designed the synthetic gene yl-HOG1-49 with the K49R mutation. Since localization of the overexpression cassette is crucial for testing the gene activity, we selected one highly active locus on chromosome E [16] and introduced all modified genes under the same promoter in the same locus.

### Reporter system for filamentous strains

In order to eliminate variability resulting from the incorporation of the expression cassette into random rDNA sequences, for the site of incorporation we chose a sequence referred to as IntE1. This is one of the available insertion sites described in the EasyCloneYALI System [16]. Although this system was optimized for *Y. lipolytica* GB20 strains, some of the sites have proven to be efficiently transcribed also in derivatives of strain A101 [20].

**Fig. 1:**
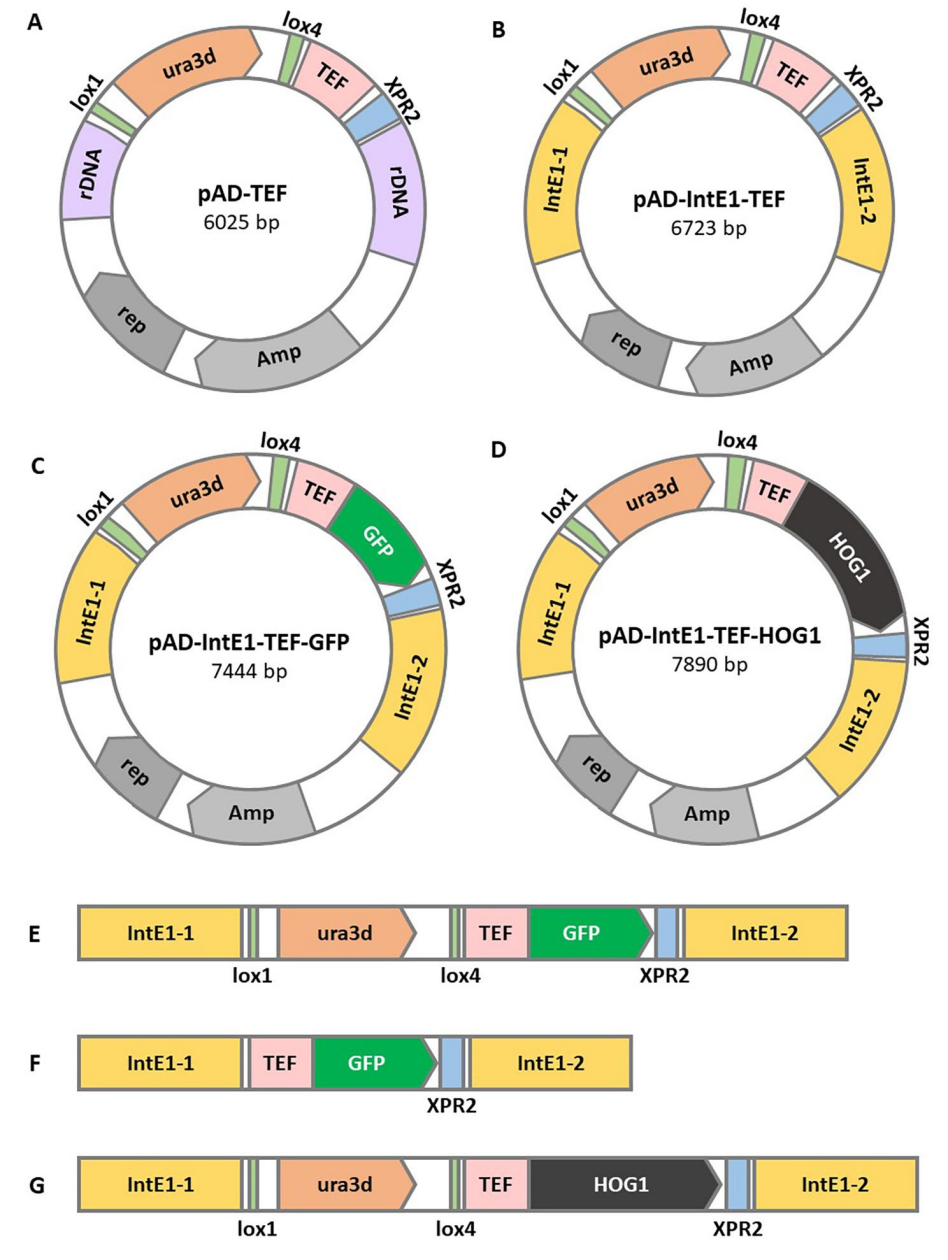
Modifications designed for the study: **A** – plasmid pAD-TEF used for further modifications [21], **B** – plasmid pAD-TEF-IntE1, **C** – plasmid pAD-IntE1-TEF-GFP, **D** – plasmid pAD-IntE1-TEF-HOG1, **E** – IntE1 region in the genome after transformation with pAD-IntE1-TEF-GFP, **F** – IntE1 region in genome with inserted GFP, after restoring auxotrophy for uracil, **G** – IntE1 region in the genome after transformation with pAD-IntE1-TEF-HOG1

Thus, a pAD-IntE1-TEF plasmid was created, which allows incorporation of the cassette into the IntE1 region by homologous recombination (Fig. 1A and B). It was inserted with the correct version of HOG1 (Fig. 1D) or the mutants HOG1-49 or HOG1-171/173. The resulting plasmids were used to transform the *Y. lipolytica yl-hog1∆* ura- strain. The selection of one specific site for incorporation into the genome required verification that the cassette was inserted into the right place. The standard method to screen for correct transformants is colony PCR, but this step is highly problematic for the yeast *Y. lipolytica.* Moreover, this proved to be particularly troublesome when working on filamentous strains. The cell wall of strain *yl-hog1∆* is more resilient to damage [14], which may have been the reason for the repetitive failures of colony PCRs. The alternative was the isolation of genomic DNA for use as a PCR template, which was more costly and time-consuming. Furthermore, isolation from *yl-hog1∆* appeared to be noticeably less efficient compared to other *Y. lipolytica* strains.

**Fig. 2:**
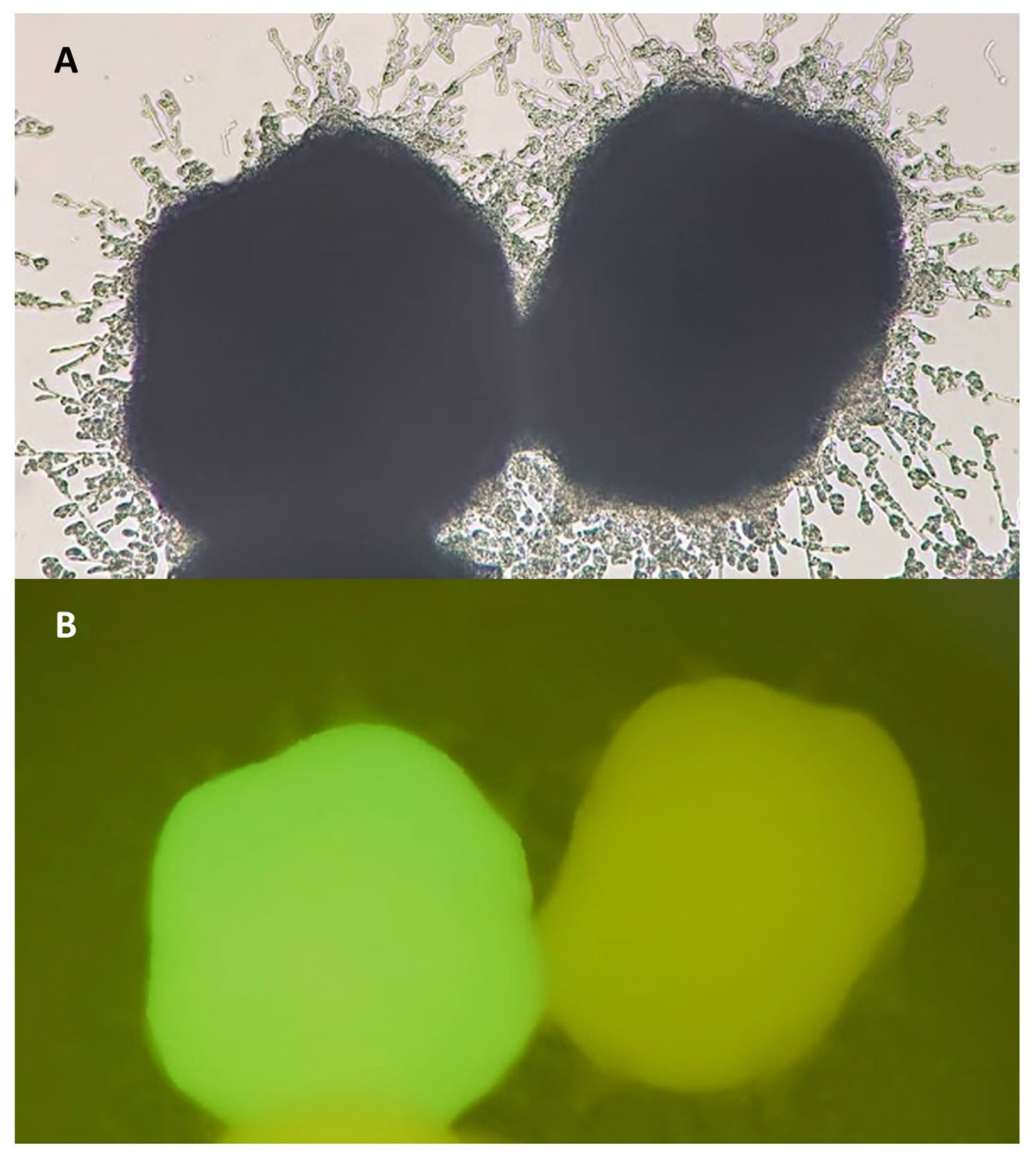
Two colonies obtained after transformation of strain AJD IntE1-GFP ura- with pAD-IntE1-TEF-Hog1: **A** – in bright field, **B** – after induction of fluorescence. The colony on the left is fluorescent, which indicates incorrect incorporation of the HOG1 cassette. The colony on the right is not fluorescent, which indicates incorporation of the cassette into the IntE1 sequence and removal of GFP

To address this issue, we created the strains AJD IntE1-GFP ura- and AJD IntE1-GFP *hog1∆* ura. They both contain the gene encoding GFP, which was incorporated into the IntE1 sequence and thus exhibits green fluorescence upon activation with 395 nm light. Moreover, both strains are auxotrophic to uracil (Fig. 1F), so they were suitable for further modifications with derivatives of the plasmid pAD-IntE1-TEF. Correct incorporation of the new expression cassette results in removal of the gene encoding GFP (Fig. 1G), and loss of fluorescence. Therefore, the initial screening of the transformants could be performed by observing the transformants under a fluorescence microscope (Fig. 2). Selected colonies, not exhibiting fluorescence, were still checked by PCR, but the initial visual screening allowed for a significant reduction in the need for genomic DNA isolation. All strains created by this method are named AJE to indicate a modifications present in IntE1 site (Table 1).

**Table 1:** Bacteria and yeast strains used in the study

Strain	Plasmid, genotype	Source
*E. coli strains*		
DH5α	F^−^ endA1 glnV44 thi-1 recA1 relA1 gyrA96 deoR nupG Φ80dlacZΔM15 Δ(lacZYA-argF)U169, hsdR17(rK-mK+), λ−	[31]
DH5α	pAD-TEF	[21]
DH5α	pUB4-Cre1(JME547)	[32]
DH5α	pQE80-pt-Hog1Ura	[14]
DH5α	pUC-Ura	[33]
DH5α	phrGFP II-C	Agilent
DH5α	pUC57-Hog1-K49R	Genewiz
DH5α	pUC57-Hog1-T171A-Y173A	Genewiz
DH5α	pAD-IntE1-TEF	This study
DH5α	pAD-IntE1-TEF-GFP	This study
DH5α	pAD-IntE1-TEF-HOG1	This study
DH5α	pAD-IntE1-TEF-HOG1-K49R	This study
DH5α	pAD-IntE1-TEF-HOG1-T171A-T173A	This study
DH5α	pUC-Ura-p-C04928	This study
DH5α	pUC-Ura-pt-C04928	This study
*Y. lipolytica strains*		
AJD	MATA, A101: ura3-302	[34]
AJD	*hog1∆*	[14]
AJD	*hog1∆* ura-	[14]
AJD	IntE1-GFP	This study
AJD	IntE1-GFP ura-	This study
AJD	IntE1-GFP *hog1∆*	This study
AJD	IntE1-GFP *hog1∆* ura-	This study
AJE	ura+, IntE1	This study
AJE	HOG1	This study
AJE	HOG1-49	This study
AJE	HOG1-171/173	This study
AJE	*hog1∆* HOG1	This study
AJE	*hog1∆* HOG1-49	This study
AJE	*hog1∆* HOG1-171/173	This study
AJD	IntE1-GFP *hog1∆ ypd1∆*	This study
AJD	IntE1-GFP *hog1∆ ypd1∆* ura-	This study
AJD	IntE1-GFP *hog1∆ ypd1∆* HOG1-49	This study
AJD	IntE1-GFP *hog1∆ ypd1∆* HOG1-171/173	This study

### Sensitivity of the engineered *Y. lipolytica* to osmotic stress

**Fig. 3:**
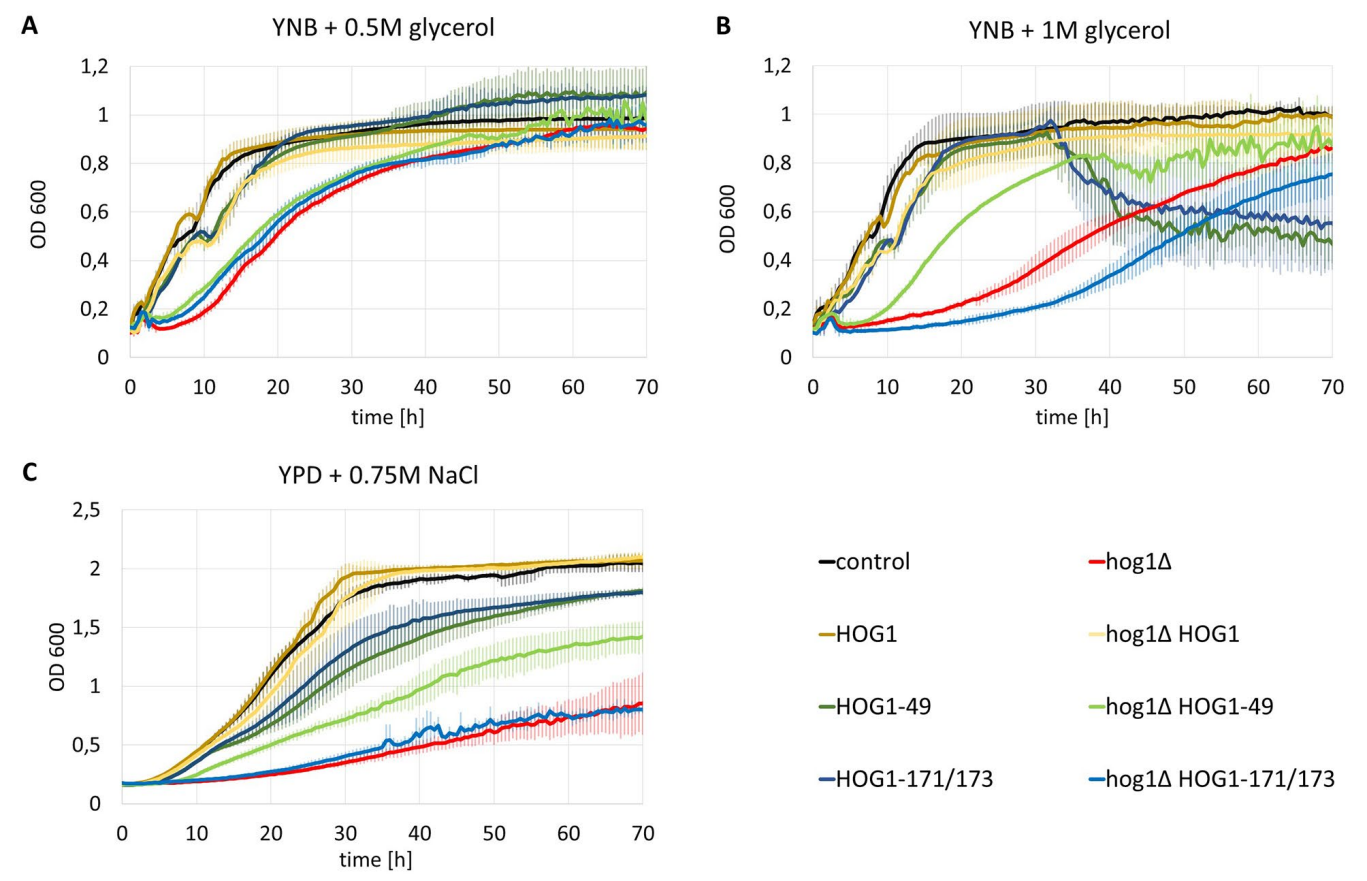
Sensitivity of *Y. lipolytica* AJE strains to osmotic stress test in liquid culture, performed in 96-well plates. **A** – YNB + 0.5 M glycerol medium, **B** – YNB + 1 M glycerol, **C** – YPD + 0.75 M NaCl medium. Error bars are indicated by semi-transparent coloration. The experiment was performed in quadruplicate

The first step in evaluating the effects of the mutations was to test the capacity for growth under osmotic stress. The study was carried out in a 96-well plate under continuous shaking at 28 °C. In two of the tested conditions YNB medium was used, and the osmotic stress was induced solely by a high concentration of the carbon source, which was glycerol (Fig. 3A and B). In the third culture, a rich YPD medium was used, and stress was induced by the addition of NaCl in a concentration of 0.75 M (Fig. 3C).

The main goal of the culture was to see how different variants of yl-HOG1 could complement the loss of the gene at its native locus (*yl-hog1∆*). When cultured in medium with 0.5 M glycerol, the strains *yl-hog1∆*, *yl-hog1∆* HOG1-49 and *yl-hog1∆* HOG1-171/173 grew in a similar manner – they showed a noticeable lag compared to the control, but ultimately achieved comparable OD values (Fig. 3A). The changes were more pronounced with 1 M glycerol, which causes higher osmotic pressure. In this case, strain *yl-hog1∆* HOG1-49 grew better than *yl-hog1∆*, although still more weakly than the control (Fig. 3B). On the other hand, strain *yl-hog1∆* HOG1-171/173 proved to be even more sensitive to stress than *yl-hog1∆*. Better growth of *yl-hog1∆* HOG1-49 was also noted on the medium YPD + 0.75 M NaCl. In this case, however, there was no clear difference between *yl-hog1∆* and *yl-hog1∆* HOG1-171/173, which were both very sensitive to stress.

Additionally, it was tested whether overexpression of the modified genes could have a negative effect on strains not previously subjected to deletion. These speculations proved to be right. Growth of strains carrying both the native gene and additional yl-HOG1-49 or yl-HOG-171/173 differed from the control. In the case of medium with 1 M glycerol (Fig. 3B), there was a clear decrease in OD of HOG1-49 and HOG1-171/173 at 32 h of culture. That could indicate a change in the growth form of the cells. On medium YPD + 0.75 M NaCl, strains HOG1-49 and HOG1-171/173 showed lower OD_600_ growth compared to the control (Fig. 3C). Such problems were not observed when the wild-type version of HOG1 was overexpressed. We suspect that the damaged versions of the protein competed with the normal form in the cell, resulting in greater stress sensitivity.

### Erythritol metabolism in Hog1 mutants

**Fig. 4:**
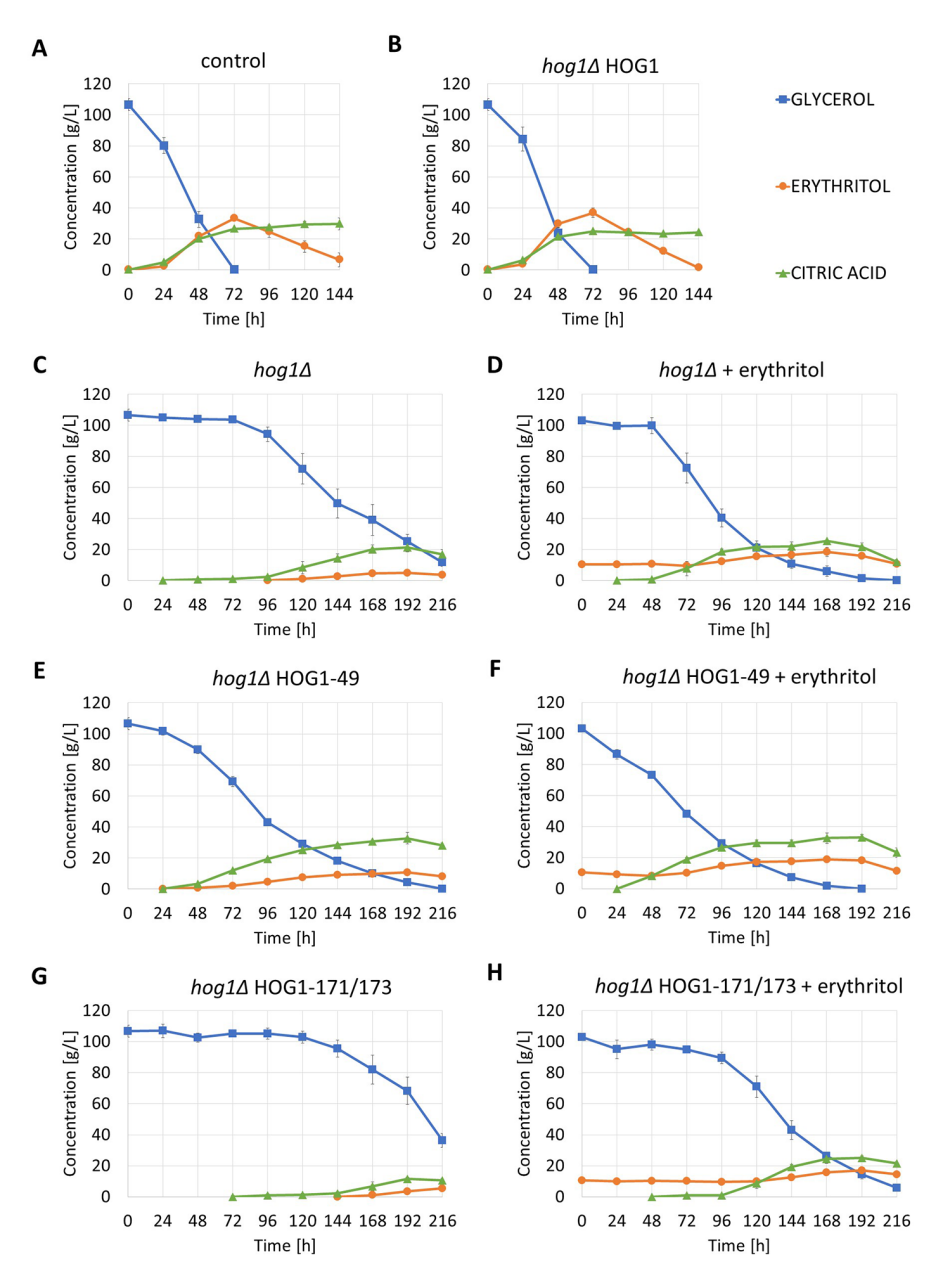
Growth of *Y. lipolytica* AJE strains on Erythritol Synthesis Medium **A**, **B**, **C**, **F**, **G** – pure ESM medium, **D**, **F**, **H** – ESM medium with erythritol supplementation

A characteristic element of *Y. lipolytica*’s response to osmotic stress is erythritol production. The medium for erythritol production (ESM) [22] contains significant amounts of glycerol (100 g/L – 1.08 M) and also addition of the salt NaCl (25 g/L – 0.42 M). Strains with a functional HOG pathway are able to grow and rapidly utilize glycerol (Fig. 4A and B). After 72 h of culture, the glycerol is depleted and erythritol reaches maximum concentration (33.3 ± 1.7 g/L for AJD and 36.9 ± 3.1 g/L for *hog1∆* HOG1). The culture was completed after 144 h, when almost all the erythritol had been re-assimilated, although large amounts of the by-product citric acid remained.

The *hog1∆* strain was initially unable to grow on ESM medium – it was not until around 96 h when it adapted to the conditions sufficiently to start consuming glycerol (Fig. 4C). However, the utilization was very slow, and when the culture ended after 216 h, glycerol was still present in the medium. The production of erythritol was also very low, reaching only 4.9 ± 1.7 g/L. This result indicates that erythritol production might be activated by the HOG pathway, and once it is damaged, the production is much less efficient. However, a second reasoning could be that osmosensitive strains are unable to produce erythritol, because they can barely grow and consume glycerol on ESM medium. A way to test these possibilities may be to improve the ability of the strain *hog1∆* to grow under severe osmotic stress. The best osmoprotectant for *Y. lipolytica* is erythritol itself [3]. Unquestionably, supplementing the ESM medium with erythritol does not have application validity, but it may be beneficial for better understanding the mechanism of the stress response. The addition of 10 g/L erythrol to the *hog1∆* culture advanced the initiation of glycerol utilization by as much as 48 h (Fig. 4D), but did not significantly increase erythrol production – the difference between its maximum and initial concentrations did not exceed 8 g/L. This further indicated the key role of yl-HOG1.

The study of *hog1∆* HOG1-49 provides an opportunity to determine the relevance of yl-HOG1 phosphorylation activity for erythritol production (Fig. 4E). This strain performed significantly better than *hog1∆* on EPM medium. Glycerol utilization started without a delay, although it noticeably slowed down after 96 h and glycerol was completely depleted only at 216 h. Erythritol production was twice higher than for *hog1∆* and reached a concentration of 10.6 ± 0.5 g/L, but this was still three times lower than for the control. The contrast was the production of citric acid, whose maximum concentration was 32.7 ± 3.7 g/L and was even higher than the control (29.7 ± 3.9 g/L). Erythrol supplementation (Fig. 4F) to the *hog1∆* HOG1-49 culture further accelerated glycerol utilization and citric acid production, but again did not improve erythritol production.

The strain *hog1∆* HOG1-171/173 was again proved to be even more sensitive to osmotic stress than *hog1∆*. Glycerol depletion was only noticeable at 144 h of culture. Erythrol supplementation shortened the time required for adaptation, but it was still as high as 96 h.

### Differences in growth form of *Y. lipolytica* HOG1 mutants

**Fig. 5:**
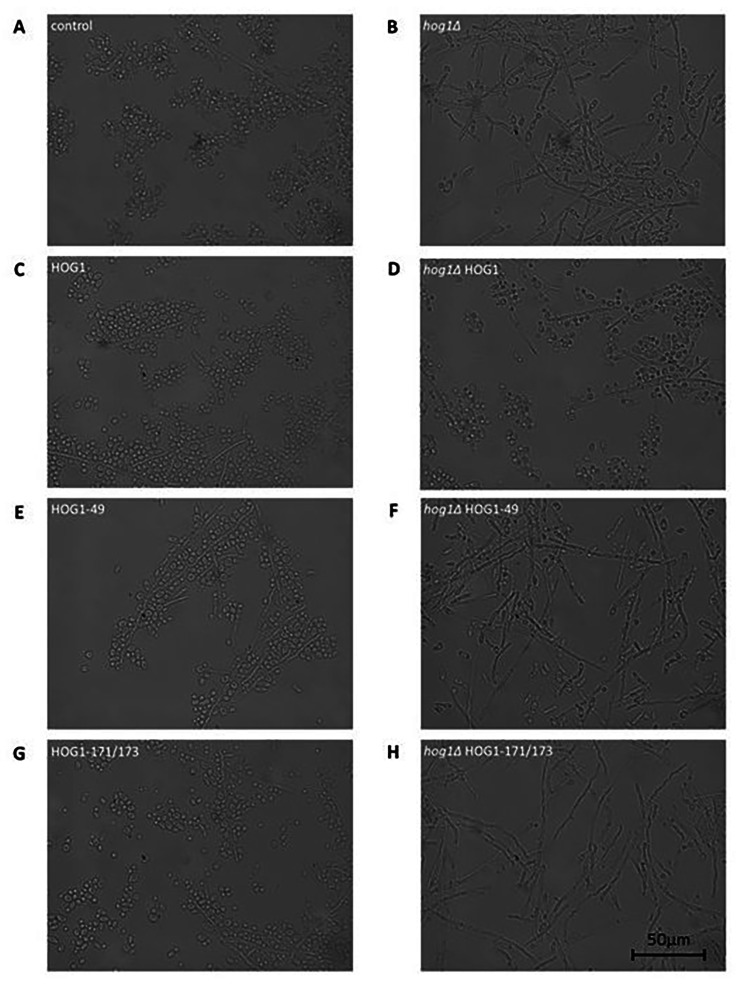
Microscope images of *Y. lipolytica* AJE cells after 24 h of growth on liquid YPD medium

The last tested aspect of K49R and T171A-Y173A point mutations was their impact on growth form. When the HOG pathway was not subjected to any modification, the yeast-like growth form was substantially dominant in YPD medium (Fig. 5A). Loss of yl-Hog1 resulted in significant prevalence of the filamentous or atypically shaped cells (Fig. 5B). On the other hand, modifications such as complementation of deletion (Fig. 5D), or overexpression of additional yl-HOG1 variants also resulted in the observation of a higher number of filamentous cells (Fig. 5C/E/G). This was particularly pronounced in the case of strain HOG1-49. This indicates that any type of HOG pathway perturbation can affect cross-talk between signaling pathways.

However, the most relevant result is the strong filamentation of strains *hog1∆* HOG1-49 and *hog1∆* HOG1-171/173 (Fig. 5F and H). In both cases, we found that expression of mutant versions of HOG1 did not allow recovery of the yeast-like growth form.

### Constant activation of HOG pathway

The last element of investigating the activation of yl-Hog1 would be to determine the effect of its constant activation. To test this, we did not introduce point mutations, but as a target for modification we chose the protein yl-Ypd1, which controls the MAPK cascade. In *S. cerevisiae*, Ypd1 phosphorylates the MAPKKK element of the cascade, resulting in its deactivation. Under osmotic stress conditions, the phosphorylation activity of Ypd1 is stopped, which in turn activates the cascade and eventually leads to Hog1 phosphorylation. Thus, knock-out of Ypd1 leads to hyperactivation of the HOG pathway, which proves to be lethal under many conditions [23].

Based on BLAST analyses, we identified the *YALI0C04928g* gene as putatively encoding yl-Ypd1. We attempted to perform a gene deletion, using the plasmid pUC-Ura-ptC0428, but it repeatedly failed. We considered the possibility that, similarly as in *S. cerevisiae*, continuous activation of the HOG pathway may be lethal in *Y. lipolytica*. However, the unsuccessful deletion was not enough to draw a clear conclusion.

In order to further investigate this topic, we performed a number of modifications, which are shown in Table 2. First of all we repeated the deletion procedure, but this time on a strain with the knock-out of yl-Hog1 (AJD IntE1 GFP *hog1∆* ura-). It was possible to obtain the strain bearing both *ylhog1∆ yl-ypd1∆* deletions. The strain with the double deletion was further transformed with the plasmid pAD-IntE1-TEF-HOG1 but no colonies were obtained. To ensure that this result was not the effect of an error in the procedure—an incorrect plasmid or improper preparation of competent cells—both of these prepared elements were used simultaneously in parallel transformations. The AJD IntE1 *hog1∆ ypd1∆* ura- competent cells were successfully transformed with defective forms of HOG1-49 or HOG1-171/173, while the pAD-IntE1-TEF-HOG1 was used in transformation of strains AJD IntE1 GFP ura- and AJD IntE1 GFP *hog1∆* ura- (Table 2). The combination of these results indicates that deletion of yl-Ypd1 cannot be performed without prior damage to the HOG pathway; thus continued activation of yl-Hog1 is lethal.

**Table 2:** Summary of investigation of activation of yl-Hog1

		Transformed strains		
		AJD IntE1-GFP ura-	AJD IntE1-GFP *hog1∆* ura-	AJD IntE1-GFP *hog1∆ ypd1∆* ura-
		obtained strains		
**Plasmids used for transformation**	pUC-Ura-ptC0428 (deletion of Ypd1)	AJD IntE1-GFP *ypd1∆* **FAILED** (No deletion transformant found)	AJD IntE1-GFP *hog1∆ ypd1∆* SUCCEED	-
	pAD-IntE1-TEF-HOG1 (Overexpression of Hog1)	AJE HOG1 SUCCEED	AJE *hog1∆* HOG1 SUCCEED	AJE *hog1∆ ypd1∆* HOG1 **FAILED** (No colonies after transformation)
	pAD-IntE1-TEF-HOG1- K49R (Overexpression of Hog1-49)	AJE HOG1-49 SUCCEED	AJE *hog1∆* HOG1-49 SUCCEED	AJE *hog1∆ ypd1∆* HOG1-49 SUCCEED
	pAD-IntE1-TEF-HOG1-T171A-T173A (Overexpression of Hog1-171/173)	AJE HOG1-171/173 SUCCEED	AJE *hog1∆* HOG1-171/173 SUCCEED	AJE *hog1∆ ypd1∆* HOG1-171/173 SUCCEED

## Discussion

The Hog1 protein is highly conserved among yeasts, so the introduction of point mutations in yl-Hog1 resulted in similar results to those obtained previously for *S. cerevisiae*. However, due to the dual nature of *Y. lipolytica*, we had found a new function of Hog1 protein from yeast to filamentous hyphae transition in this yeast. The strain *hog1∆* HOG1-171/173 was found to be extremely sensitive to osmotic stress, which is consistent with observations from the yeast *S. cerevisiae* [24]. This indicates a key role of the protein’s phosphorylation capacity for its ability to survive in a hyperosmotic environment. Interestingly, this strain was found to be even more sensitive to osmotic stress than *hog1∆*. We suspect that this may be the effect of additional burden on the cell by the production of a non-functional protein. The strain *hog1∆* HOG1-49 coped significantly better with osmotic stress than *hog1∆*. This was again a very similar result to *S. cerevisiae* strains that have a lysine to arginine mutation in the active site. This mutation, despite severely damaging the active site, was shown to still allow residual activity, improving growth under stress conditions [23]. An interesting result is the observation of filamentation. Another conservative property of Hog1 in yeast is its negative regulation of other MAPK signaling pathways [23]. In the case of the dimorphic yeast *Y. lipolytica*, the interaction with the pathway controlling the change in growth form is particularly important. The abnormally elevated filamentation of the strain *hog1∆* under a variety of culture conditions indicates that Hog1 activity is important for maintaining the yeast growth form even when the HOG pathway is not activated by osmotic stress [14]. This points to an important role of yl-Hog1 basal activity. The basal activity of MAPK cascades means that a small fraction of these proteins is constantly active to maintain a balance between different signaling pathways, but also to accelerate the activation of the stress response when needed [25]. The fact that both strains *hog1∆* HOG1-49 and *hog1∆* HOG1-171/173 show a very similar form of growth to *hog1∆*, enables two conclusions to be drawn about the yl-Hog1 basal activity. First, any potential residual activity that enhances survival of strain *hog1∆* HOG1-49 is not sufficient to maintain negative control over other MAPK pathways. Second, the basal activity of yl-Hog1 depends on its phosphorylation. The variant of yl-HOG1 with mutations targeting T171 and Y173 is inactive despite having a functional enzyme center.

The enzymatic activity of yl-Hog1 is also important for the induction of erythrol synthesis. In earlier studies, we considered whether the very low erythritol production by the *hog1∆* strain is caused primarily by poor growth on ESM medium. The less osmosensitive *hog1∆* HOG1-49 does not exhibit a long lag phase and quickly begins glycerol utilization, but this does not translate into much higher erythritol production. Indeed, the maximum concentration for the *hog1∆* HOG1-49 strain is 10.6 ± 0.5 g/L, which is twice that of *hog1∆*, but still much lower than for the control (33.3 ± 1.7 g/L). Moreover, it has already been observed that erythritol production by the strain *hog1∆* could also reach 10 g/L [14]. Mutations in the Hog1 protein do not affect the osmoprotective effect of erythritol on cells. An improvement in growth due to erythritol supplementation was evident for both *hog1∆* Hog1-49 and *hog1∆* HOG1-171/173, though the effect was more pronounced for the more sensitive strain.

Finally we wanted to investigate the effect of continuous phosphorylation of yl-Hog1. It was expected that preparation of transformants might be difficult or impossible. Activation of the HOG pathway causes a temporary arrest of the cell cycle, until the cell adapts to the higher osmotic pressure [13].

Hyperactivation of Hog1 might help in adaptation to some stressors – like presence of 2-phenylethanol [26].

However, too long activation of HOG might be dangerous for the cell, and tight control is necessary. Loss of Ypd1, which inhibits HOG activity in the absence of stress, has been shown to be lethal for *S. cerevisiae*. Additional modifications, such as removal of elements of the MAPK cascade, were necessary to obtain *sc-ypd1∆* strains [27]. In contrast, the yeast *C. albicans* was able to survive the Ypd1 deletion, although it strongly impairs growth [28]. Our results indicate that in *Y. lipolytica* loss of Ypd1 also is lethal. All this showed that the role of yl-Hog1 protein in erythritol synthesis still needs to be elucidated, and inactivation of both phosphorylation and the active center of yl-HOG proteins leads to impaired erythritol synthesis.

Hyperosmotic conditions can negatively impact some biotechnology applications of *Y. lipolytica* – such as synthesis of recombinant proteins [29]. Better understanding of mechanisms behind osmotic stress response might contribute to easier optimization of industrial processes. Moreover, shift of growth toward filamentous form by targeting the active center of yl-Hog1 might unlock a new applications of this non-conventional yeast.

## Conclusions

In dimorphic yeast the filament-to-yeast transition is an important process that can be induced by environmental factors such as carbon limitation, nitrogen starvation or low oxygen or transcriptional factors. In this study, we showed that yl-Hog1 protein is also involved in this process in yeast *Y. lipolytica*. Point mutations in the active center or in phosphorylation site of yl-Hog1, significantly impaired its function. Deletion of Ypd1 protein, which controls the MAPK cascade, in the strain with active yl-Hog1 protein, is lethal for cells. Moreover, here we showed that the *hog1∆* HOG1-49 strain is able to grow under elevated osmotic pressure caused by high concentration of glycerol and displays filamentous growth, which might be better suited to specific industrial fermentation purposes.

## Methods

### Microorganisms

The strain used in the study was *Yarrowia lipolytica* AJD, the ura3∆ derivative of *Y. lipolytica* A101 [30]. *Y. lipolytica* AJD was subjected to the deletion of protein ylHog1, resulting in the *yl-hog1∆* strain and further modifications described in the following sections. The strains derived from the AJD strain were named AJE, which indicate modifications incorporated into the IntE1 integration site.

Strains of *Escherichia coli* DH5α were the carriers of plasmids containing deletion or overexpression cassettes and were used for transformation procedures. The full list of used and created strains and plasmids is presented in Table 1.

### Media and culture conditions

*E. coli* strains used in the transformation procedures were cultivated in LB medium (A&A Biotechnology, Poland). The antibiotic used as a marker was ampicillin (50 µg/mL).

YPD medium (A&A Biotechnology, Poland) was used for yeast inoculum preparation. YNB without amino acids (Merck, Germany) agar plates with 2% (w/v) glucose (Chempur, Poland) were used for standard yeast transformations. YPD agar plates with Hygromycin B (800 µL/100 mL) were used for yeast transformations to restore auxotrophy. YPD media with different amounts of NaCl (Chempur, Poland) were used in stress sensitivity tests.

YNB media with varying concentrations of glycerol (Wratislavia-Biodiesel, Poland) were also used in stress sensitivity tests. The carbon sources were: glucose (Chempur), glycerol, or erythritol (Młyn Oliwski, Poland). Glycerol, glucose, and erythritol used in the media were sterilized by filtration and added to other compounds, just before the preparation of the agar plates. All yeast strains were cultivated at 28 °C.

The test of sensitivity to osmotic stress was performed in 96-well plates. 200 µL of medium in each well was inoculated to an OD_600_ value of 0.15 (which is around 9 × 10^5^ cells/mL for both yeast and filamentous growth form). Quintuple experiments were performed at 28 °C under constant agitation with a Tecan system. Growth was monitored by measuring the optical density at 600 nm every 30 min for 72 h.

The ability of yeasts to synthesize erythritol was tested on Erythritol Synthesis Medium (ESM) containing 100 g/L glycerol, 2.3 g/L (NH_4_)_2_SO_4_ (Chempur), 1 g/L MgSO_4_ × 7H_2_O (Chempur), 0.23 g/L KH_2_PO_4_ (Chempur), 26.4 g/L NaCl (Chempur), 1 g/L yeast extract (A&A Biotechnology) and 3 g/L CaCO_3_, pH 3.0. CaCO_3_ was added separately to each flask after establishing pH 3 in order to prevent a fall of pH value. Optionally 10 g/L of erythritol (Młyn Oliwski, Poland) was also added to the ESM medium. Cultures were grown in 0.3 L flasks with baffles containing 0.05 L of medium kept on a rotary shaker at 240 rpm.

### Cloning and bacteria transformation protocols

All restriction enzymes were purchased from FastDigest Thermo Scientific and all of the digestions were performed according to standard protocols. The PCR reactions were set up using recommended conditions and high-fidelity Phusion DNA polymerase (Thermo Scientific). The ligation reactions were performed for 10 min at room temperature using T4 DNA Ligase (Thermo Scientifc). The gel extractions were performed using the Gel Out extraction kit purchased from A&A Biotechnology (Poland). The *E. coli* minipreps were performed using the Plasmid Mini Kit (A&A Biotechnology). Transformation of *E. coli* strains was performed using standard chemical protocols [31]. A list of all primers used in the study is presented in Table 3.

**Table 3:** Primers used in the study. Underline indicates presence of restriction site

Name	Sequence	Purpose
A-NcoI-F	ATACCATGGAATTATCGCTTCGGATAACTC	Creating plasmid pAD-IntE1-TEF
A-BamHI-R	GCTGGATCCCTCATTTGAATATTTGCTACTAC	
IntE1-1-BamHI-F	ATAGGATCCGAGGCGACACTGTTGATTGC	
IntE1-1-NcoI-R	ATTCCATGGCGCGAAAGTCCACTAATGGC	
IntE1-2-SphI-F	TATGCATGCGCAGCAGAGGATAGTGCTTGTG	
IntE1-2-HindIII-R	GATAAGCTTGCGTTCAGAGGGCTTTTGGAGAC	
hrGFP-AscI-F	TATGGCGCGCCATGGTGAGCAAGCAGATC	Creating plasmid pAD-IntE1-TEF-GFP
hfGFP-NheI-R	GCAGCTAGCCTGCAGAATTCCTATTAC	
Hog1-AscI-F	AATGGCGCGCCTAACATGGCGGACTTTATC	Creating plasmid pAD-Inte1-TEF-HOG1
Hog1-Nhe1-R	AGAGCTAGCACATTGTTTGGGTGTTTACTG	
pp-IntE1	GACACGATCCAGCGATAG	Testing an integration of cassettes into IntE1 region
IntE1-2-col-R	GAGGGCTGTATGCTTGTC	
TEF-col-R	CGCTACTGGGTCAATTTGG	
p4928-SalI-F	ATTGTCGACCAAATACCAGCGATTAGTCAC	Creating yl-Ypd1 deletion cassette
p4928-ApaI-R	ATAGGGCCCAGATGTGCCTCTTCGATTTC	
t4928-NotI-F	ATAGCGGCCGCTTTGCCAAATGAACAACTTCC	
t4928-PmeI-R	CTGGTTTAAACTCACCGTGTTAATGTTGACTC	

### Plasmid pAD-IntE1-TEF

A base for the preparation of the new plasmids was pAD-TEF (Fig. 1A), which carries the cassette containing the TEF promoter [35], XRP2 terminator and gene Ura3d between motifs lox1 and lox4 [32]. The cassette is flanked by rDNA sequences that allow integration into the yeast ribosomal DNA region [15]. In this study, we wanted to change the integration site from the rDNA sequence to the IntE1 region [16]. It was done in two steps.

The first part of the IntE1 sequence was replicated from *Y. lipolytica* genomic DNA with the primers IntE1-1-BamHI-F and IntE1-1-NcoI-R, creating the insert IntE1-1. The plasmid pAD-TEF was a template for PCR reaction with A-NcoI-F and A-BamHI primers. The product contained most of the pAD-TEF sequence except one of the rDNA parts. These two fragments were digested by NcoI and BamHI enzymes and ligated, resulting in the intermediate plasmid pAD-TEF-rDNA-IntE1-1. Next, digestion of this plasmid with the enzymes SphI and HindIII removed the second rDNA region. The fragments were separated by electrophoresis, and the pAD-TEF-IntE1-1 backbone isolated from the gel was used ligated with the insert IntE1-2. The IntE1-2 fragment was earlier obtained by PCR on genomic DNA using the primers IntE1-2-SphI-F and IntE1-2-HindIII-R and digestion by the enzymes SphI and HindIII. The resulting plasmid is pAD-IntE1-TEF (Fig. 1B).

### Plasmid IntE1-TEF-GFP

The GFP insert was replicated from the commercial plasmid phrGFP II-C (Agilent) using the primers hrGFP-AscI-F and hrGFP-NheI-R. The resulting PCR product and the pAD-TEF-IntE1 plasmid were digested with AscI and NheI enzymes and then ligated to form the IntE1-TEF-GFP plasmid (Fig. 1C).

### Variants of HOG1 gene

The native yl-Hog1 gene (*YALI0E25135g*) was replicated from genomic DNA using the primers Hog1-AscI-F and Hog1-Nhe1-R. The PCR product was digested with AscI and NheI enzymes and inserted into pAD-TEF-IntE1. The resulting plasmid was named IntE1-TEF-Hog1 (Fig. 1D).

Two modified variants of the Hog1 gene were ordered as synthetic genes from GeneWiz and delivered on plasmids pUC57-Hog1-K49R and pUC57-Hog1-T171A-Y173A. The genes were amplified by PCR reaction, also with primers Hog1-AscI-F and Hog1-Nhe1-R. After digestion with the enzymes AscI and NheI they were integrated into pAD-IntE1-TEF. The resulting plasmids are named pAD-IntE1-TEF-Hog1-K49R and pAD-IntE1-TEF-Hog1-T171A-Y173A.

### Yl-Ypd1 deletion cassette

Promotor and terminator sequences of the gene *YALI0C04928g* encoding yl-Ypd1 were replicated using the primers p4928-SalI-F and p4928-ApaI-R (promoter), t4928-NotI-F and t4928-PmeI-R (terminator). The promoter sequence and pUC-Ura plasmid were digested by the enzymes SalI and ApaI and ligated into the plasmid pUC-Ura-pC04928. This plasmid and terminator sequence were digested with NotI and PmeI and ligated into the plasmid pUC-Ura-ptC0428.

### Yeast transformation

For transformations of *Y. lipolytica*, strains with auxotrophy for uracil were used. Transformations were performed according to the lithium acetate method[33] and transformants were plated out on selective media without uracil (YNB + 2% glucose). They were analyzed for proper integration by gDNA extraction and PCR amplification with two primer pairs. Genomic DNA (gDNA) was extracted from *Y. lipolytica* using the GeneMATRIX Bacterial & Yeast Genomic DNA Purification Kit (EURx, Poland).

A particular type of transformation was the recovery of auxotrophy to uracil. The replication plasmid pUB4-Cre1[29] was used, and transformants were planted on YPD medium with Hygromycin B. Colonies were subjected to three passages on YPD with Hygromycin B plates to recover auxotrophy, followed by passages on YPD plates until the replication plasmid was removed.

In order to facilitate the screening of transformants after insertion of overexpression cassettes, a special AJD IntE1-GFP ura- strain was created. This required first transforming the AJD ura- strain with the pAD-IntE1-TEF-GFP plasmid to insert the GFP gene into the IntE1 region (Fig. 1E). In order for this strain to be used in further transformations, it underwent recovery of auxotrophy for uracil (Fig. 1F). The proper integration was tested by multiple PCR reactions (primers pp-IntE1, IntE1-2-col-R, TEF-col-R) The second relevant strain was AJD IntE1-GFP *hog1∆* ura-, created by transforming AJD IntE1-GFP ura- with the plasmid pQE80-pt-Hog1Ura [14], and recovering auxotrophy. Both strains showed green fluorescence due to the GFP gene in the IntE1 region. After transformation with the plasmids pADIntE1-TEF-HOG1, pAD-IntE1-TEF-HOG1-49 or pAD-IntE1-TEF-HOG1-171/173, fluorescence was lost when the cassette was incorporated into the correct IntE1 site (Fig. 1G). Thus the first step of screening of proper integration for the strain with the GFP reporter system was microscopic observation.

### Microscopy

Differential interference contrast (DIC) images of yeast cells were captured using a Zeiss Scope A1 Microscope using a Plan-Neofluar 40x objective. Images were taken of the indicated strains following 24 h growth in YPD medium. Microscopy data were stored using ZEN lite (Zeiss). The GFP signal was used for screening of *Y. lipolytica* transformants. Agar plates were observed using the 5x objective. The GFP filter set (Zeiss), operated at excitation of 470/40 nm, and emission of 525/50 nm, was used to detect green fluorescence.

### Analytical methods

The concentrations of polyols and organic acids in cultures were determined by HPLC using a HyperRez Carbohydrate H + Column (Thermo Scientific, Waltham, MA, USA) coupled to a UV detector (λ = 210 nm) (Dionex, Sunnyvale, CA, USA) and a refractive index detector (Shodex, Ogimachi, Japan). 0.25% trifluoroacetic acid was used as a mobile phase solvent. The samples were diluted 10-fold before the measurement. Data were analyzed with the Chromeleon program.

### Statistical analysis

Sensitivity tests were performed in four biological repetitions, cultures for erythritol production were performed in three repetitions. Obtained values were used for calculation of means and standard errors.

In erythritol production experiments if necessary, the differences between two data sets were evaluated by t-Student’s test for independent samples.
